# A central role for a region in the middle

**DOI:** 10.7554/eLife.25700

**Published:** 2017-03-07

**Authors:** Edward Stuttfeld, Stefan Imseng, Timm Maier

**Affiliations:** Biozentrum, University of Basel, Basel, Switzerland; Biozentrum, University of Basel, Basel, Switzerland; Biozentrum, University of Basel, Basel, Switzerlandtimm.maier@unibas.ch

**Keywords:** Target of Rapamycin, SIN1, AKT, substrate specificity, TOR complex 2, TOR signaling, *S. pombe*, Human

## Abstract

A domain called the 'Conserved region in the middle' is responsible for target recognition in the TORC2 complex in fission yeast and the mTORC2 complex in mammals.

**Related research article** Tatebe H, Murayama S, Yonekura T, Hatano T, Richter D, Furuya T, Kataoka S, Furuita K, Kojima C, Shiozaki K. 2017. Substrate specificity of TOR complex 2 is determined by a ubiquitin-fold domain of the Sin1 subunit. *eLife* **6**:e19594. doi: 10.7554/eLife.19594

Cellular growth is a tightly regulated process that depends on the availability of energy and nutrients. Moreover, in multicellular species, the proliferation of cells must be coordinated across tissues and organs. Protein kinases called target of rapamycin (TOR) proteins and their mammalian ortholog mTOR have a central role in growth regulation. These proteins exert their function in two complexes called TORC1 and TORC2 (or mTORC1 and mTORC2 in mammals). These complexes act as regulatory hubs that integrate input signals concerning the availability of energy and nutrients or the presence of growth factors. With their outputs, the mTOR complexes control metabolism and protein biosynthesis, and influence cell cycle progression, autophagy and cytoskeletal organization (Laplante and Sabatini, 2012; Shimobayashi and Hall, 2014).

TOR and mTOR are very large proteins (containing approximately 2300–2600 amino acids) and are members of the PIKK family of regulatory kinases. Both mTOR complexes comprise mTOR and a protein called mLST8 (Figure 1A). mTORC1 also contains a protein called Raptor, whereas mTORC2 further includes the proteins Rictor and SIN1. In both complexes, the partner proteins of mTOR determine the target specificity of the mTOR kinase (Cameron et al., 2011; Nojima et al., 2003). mTORC1 phosphorylates a diverse set of targets involved in protein biosynthesis, metabolism and transcriptional regulation, while mTORC2’s most prominent targets are regulatory kinases of the AGC family. [Fig fig1]

**Figure 1. fig1:**
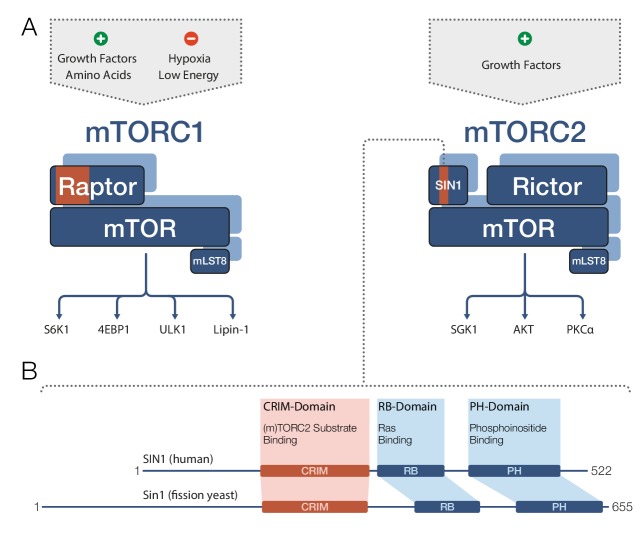
Target recognition in mTOR complexes. (**A**) The protein complexes mTORC1 and mTORC2 regulate cellular processes and growth via phosphorylation of substrate proteins mediated by the mTOR kinase domain. mTORC1 signaling (left) is stimulated by growth factors and amino acids, and is inhibited by low cellular energy levels and hypoxia (a shortage of oxygen). Conserved protein regions involved in target recognition in mTORC1 are located in the Raptor subunit (highlighted in orange). mTORC2 signaling (right) is activated by growth factors and the substrate binding domain identified by Tatebe et al. – the 'Conserved region in the middle' (CRIM) domain – is located in the SIN1 subunit (orange). Prominent downstream targets for each complex are indicated, including mTORC2 targets from the AGC kinase family: SGK1, AKT and PKCα. (**B**) The human SIN1 protein (top) and its fission yeast ortholog Sin1 (bottom) share similar domain organizations. The CRIM domain is involved in target recognition in both mTORC2 and TORC2. The variable N-terminal domain (which is to the left of the CRIM domain) has been implicated in SIN1/Sin1’s association with other constituents of mTORC2/TORC2. mLST8: mammalian lethal with SEC13 protein 8; PH: pleckstrin-homology; PIKK: phosphoinositide-3-kinase-related kinase; Raptor: regulatory-associated protein of mTOR; RB: (Raf-like) RAS-binding; Rictor: rapamycin-insensitive companion of mTOR; Sin1/SIN1: stress-activated map kinase-interacting protein 1.

In mTORC1, the Raptor N-terminal conserved (RNC) domain is known to be involved in the binding of linear recognition motifs in target proteins (Dunlop et al., 2009). Recent cryo-electron microscopy studies have provided a first glimpse at its function in mTORC1 (Aylett et al., 2016). The RNC domain is well positioned in the vicinity of the kinase active site to both recognize target motifs (Beugnet et al., 2003) and to sterically prevent – in cooperation with parts of mTOR – bulky non-target proteins accessing the kinase.

However, less is known about target recognition in TORC2 and mTORC2. Now, in eLife, researchers at the Nara Institute of Science and Technology, Osaka University and the University of California, Davis -- including Hisashi Tatebe and Shinichi Murayama as joint first authors, and Tatebe and Kazuhiro Shiozaki as corresponding authors -- report the results of a series of studies on human SIN1 and its fission yeast ortholog Sin1, that shed light on the conserved role of these proteins in target binding (Tatebe et al., 2017).

SIN1 and its orthologs are known to contain four domains (Figure 1B): a variable N-terminal region (Frias et al., 2006); the ‘Conserved region in the middle’ (CRIM); an RB domain; and a PH domain. However, the role of these domains in mTORC2 target recognition remained unclear, and structural data were only available for the isolated PH domain (Pan and Matsuura, 2012). Now Tatebe et al. have clearly shown that the CRIM domain has a key role in target recognition. In particular, they demonstrated that in fission yeast, the CRIM domain in Sin1 is primarily responsible for binding targets from the AGC kinase family. In human cell lines, the CRIM domain in SIN1 is at least involved in recognition of related targets, implying an evolutionary conserved role for this domain.

Based on earlier studies (Liu et al., 2015), SIN1 may even directly interact with the mTOR kinase domain in mTORC2. Thus, SIN1 might also be positioned to restrict access of the substrate to the kinase, eventually in cooperation with Rictor, similar to Raptor in mTORC1. Tatebe et al. also highlighted the crucial role of spatial proximity in target recognition by demonstrating that fusion of only the TORC2 target-binding CRIM domain into TORC1 is sufficient to let TORC1 phosphorylate a TORC2 substrate.

Tatebe et al. also report an NMR structure for the CRIM domain in fission yeast that reveals a ubiquitin-like fold with a prominent acidic loop. Mutational analysis confirms that target binding depends on the integrity of the CRIM domain and strongly suggests that the acidic loop is involved in binding.

With the functional role and structure of the CRIM domain now firmly established, researchers can start to address the many remaining questions about TORC2 target recognition. The NMR structure of the CRIM domain provides an excellent platform for a detailed analysis of the recognition of specific motifs in target substrates by SIN1. However, studies of the overall integration of SIN1 into mTORC2 will be required to address the consequences of SIN1-target binding for subsequent phosphorylation. Finally, work on target recognition in mTOR complexes together with recent structural and functional studies on other PIKK family kinases (Sibanda et al., 2017) may reveal the presence (or absence) of common principles in target recognition across the entire PIKK family.

## References

[bib1] Aylett CH, Sauer E, Imseng S, Boehringer D, Hall MN, Ban N, Maier T (2016). Architecture of human mTOR complex 1. Science.

[bib2] Beugnet A, Wang X, Proud CG (2003). Target of rapamycin (TOR)-signaling and RAIP motifs play distinct roles in the mammalian TOR-dependent phosphorylation of initiation factor 4E-binding protein 1. Journal of Biological Chemistry.

[bib3] Cameron AJ, Linch MD, Saurin AT, Escribano C, Parker PJ (2011). mTORC2 targets AGC kinases through Sin1-dependent recruitment. Biochemical Journal.

[bib4] Dunlop EA, Dodd KM, Seymour LA, Tee AR (2009). Mammalian target of rapamycin complex 1-mediated phosphorylation of eukaryotic initiation factor 4E-binding protein 1 requires multiple protein-protein interactions for substrate recognition. Cellular Signalling.

[bib5] Frias MA, Thoreen CC, Jaffe JD, Schroder W, Sculley T, Carr SA, Sabatini DM (2006). mSin1 is necessary for Akt/PKB phosphorylation, and its isoforms define three distinct mTORC2s. Current Biology.

[bib6] Laplante M, Sabatini DM (2012). mTOR signaling in growth control and disease. Cell.

[bib7] Liu P, Gan W, Chin YR, Ogura K, Guo J, Zhang J, Wang B, Blenis J, Cantley LC, Toker A, Su B, Wei W (2015). PtdIns(3,4,5)P3-Dependent activation of the mTORC2 kinase complex. Cancer Discovery.

[bib8] Nojima H, Tokunaga C, Eguchi S, Oshiro N, Hidayat S, Yoshino K, Hara K, Tanaka N, Avruch J, Yonezawa K (2003). The mammalian target of rapamycin (mTOR) partner, Raptor, binds the mTOR substrates p70 S6 kinase and 4E-BP1 through their TOR signaling (TOS) motif. Journal of Biological Chemistry.

[bib9] Pan D, Matsuura Y (2012). Structures of the pleckstrin homology domain of Saccharomyces cerevisiae Avo1 and its human orthologue Sin1, an essential subunit of TOR complex 2. Acta Crystallographica Section F Structural Biology and Crystallization Communications.

[bib10] Shimobayashi M, Hall MN (2014). Making new contacts: the mTOR network in metabolism and signalling crosstalk. Nature Reviews Molecular Cell Biology.

[bib11] Sibanda BL, Chirgadze DY, Ascher DB, Blundell TL (2017). DNA-PKcs structure suggests an allosteric mechanism modulating DNA double-strand break repair. Science.

[bib12] Tatebe H, Murayama S, Yonekura T, Hatano T, Richter D, Furuya T, Kataoka S, Furuita K, Kojima C, Shiozaki K (2017). Substrate specificity of TOR complex 2 is determined by a ubiquitin-fold domain of the Sin1 subunit. eLife.

